# Management of bilateral temporomandibular joint ankylosis using bilateral custom alloplastic temporomandibular joint prosthesis and genioplasty: A case report

**DOI:** 10.1016/j.ijscr.2023.108516

**Published:** 2023-07-20

**Authors:** Manoj Adhikari, Chandan Upadhyaya, Kanistika Jha, Galav Adhikari

**Affiliations:** a Nepalese Army Institute of Health Sciences, College of Medicine, Affiliated to Tribhuvan University, Sanobharyang, Kathmandu, Nepal; b Kathmandu University School of Medical Sciences, Dhulikhel, Nepal; c College of Medical Sciences, Affiliated to Kathmandu University, Bharatpur, Chitwan, Nepal

**Keywords:** Temporomandibular joint ankylosis, Facial asymmetry, Custom alloplastic TMJ prosthesis, TMJ replacement, Genioplasty, Case report

## Abstract

**Introduction and importance:**

Temporomandibular joint (TMJ) ankylosis can be effectively managed through the utilization of autogenous grafts or alloplastic TMJ prostheses. Alloplastic TMJ prostheses are available in two forms: stock or custom. Custom alloplastic TMJ prostheses represent an emerging treatment modality for TMJ ankylosis.

**Presentation of the case:**

A 47-year-old female patient presented with a 30-year history of complete inability to open her mouth, chew, speak, and be on a liquid diet. Bilateral TMJ ankylosis and a nine mm right-sided chin deviation were noted. A bilateral osteoarthectomy was performed, followed by reconstruction of the TMJ using a custom alloplastic TMJ prosthesis via an extended preauricular and submandibular approach. The abdominal fat pad was utilized for interposition to prevent recurrence. Genioplasty was carried out through a vestibular approach, shifting the chin nine mm to the left. Postoperatively, the patient achieved a 30 mm mouth opening, and correction of facial asymmetry resulting from chin deviation was observed.

**Clinical discussion:**

Treatment options for TMJ ankylosis include autogenous grafts and alloplastic materials. Autografts have limitations such as prolonged surgery, resorption, undergrowth/overgrowth, donor site morbidity, and graft fracture. Stock alloplastic TMJ prostheses may not suit all patients due to anatomical variations. Thus, custom alloplastic TMJ prostheses have emerged as the preferred treatment modality for adult TMJ ankylosis.

**Conclusion:**

Custom alloplastic TMJ prostheses are considered an optimal treatment modality for reconstructing the TMJ in adult patients with TMJ ankylosis.

## Highlights

• A 47-year-old female with nil mouth opening, unable to chew, speak, and on a liquid diet for 30 yrs.
• She had bilateral TMJ ankylosis and her chin deviated 9 mm to the right side.
• Extensive ankylosis on the right side: fusion of ramus and the coronoid process to the posterolateral maxilla.
• Reconstruction of TMJ was done with a custom alloplastic TMJ prosthesis and genioplasty was performed for chin correction.
• TMJ replacement surgery is prevalent in developed nations but uncommon in developing countries.

## 1 Introduction

Temporomandibular joint ankylosis (TMJA) is a pathological condition characterized by the fusion of the mandibular condyle with the temporal bone fossa resulting in a range of issues including impaired chewing, swallowing, aesthetics, oral hygiene, airway function and overall quality of life [1,2]. Trauma represents the predominant etiology for the occurrence of TMJA [[3], [4], [5]]. Autogenous grafts or alloplastic materials can be utilized as treatment modalities for TMJA [6]. The utilization of autogenous grafts in the treatment of TMJA is associated with increased surgical duration, potential resorption, donor site morbidity and the risk of graft fracture. Stock alloplastic temporomandibular joint (TMJ) prostheses have also not been universally suitable for all patients due to variations in individual bony anatomy [6]. Custom alloplastic TMJ prostheses, thus, have emerged as the preferred treatment option for adult patients with TMJA [7].

The surgery for temporomandibular joint ankylosis (TMJA) aims to achieve sufficient mouth opening, minimize the risk of recurrence, establish satisfactory occlusion, preserve ramal height, correct facial asymmetry and enhance the overall quality of life [7].

A 47-year-old female patient, who was exclusively reliant on a liquid diet, presented with a complete inability to open her mouth, chew or speak for the last 30 years. Upon examination, bilateral temporomandibular joint (TMJ) ankylosis was observed, along with a nine mm right-sided chin deviation. Notably, the extent of ankylotic involvement on the right side was exceptionally severe, as it encompassed not only the TMJ region but extended extensively to fuse the upper portion of the ramus and the coronoid process with the posterolateral surface of the maxilla. To the best of our knowledge, no previous reports have documented a case of such profound severity of TMJ ankylosis in the existing literature. The work has been reported in line with the SCARE criteria [8].

## 2 Presentation of case

**Fig. 1:**
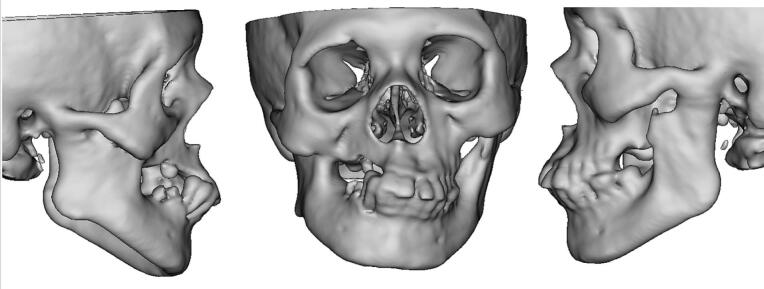
Volumetric Rendering Technique (VRT) images revealing bilateral temporomandibular joint (TMJ) ankylosis and right-sided deviation of the chin.

A 47-year-old female patient presented to the Oral and Maxillofacial Surgery Department with a chief complaint of complete inability to open her mouth for the past 30 years. The patient did not present with any significant medical or dental history, the drug history, family history, psychosocial history and genetic factors were also irrelevant. Upon extra-oral examination, bilateral TMJ movements were non-perceptible and facial asymmetry was noted with the chin deviated to the right side. Intraoral examination revealed complete limitation of mouth opening and posterior teeth loss in both jaws. Non-contrast Computed Tomography (NCCT) imaging of the face revealed the presence of ankylotic masses in both TMJ regions, with the chin deviated by nine mm to the right side (Fig. 1). Importantly, on the right side, the ankylotic mass extended beyond the TMJ area, involving the fusion of the upper portion of the ramus and coronoid process with the maxilla. Routine baseline blood investigations, electrocardiogram (ECG), chest x-ray and viral marker testing were conducted with all the results falling within the normal range.

## 3 Treatment planning

The primary treatment objectives were to restore the mouth opening and correction of the position of the chin. A comprehensive treatment plan was developed based on the Non-Contrast Computed Tomography (NCCT) imaging of the patient's face.

Considering the extent of the ankylosed region on the right side, a planned inferior osteotomy cut was proposed in the lower section of the ramus, approximately 5 mm above the mandibular foramen. Additionally, a superior osteotomy cut was planned just below the zygomatic arch. On the left side, an inferior osteotomy cut was planned at the base of the sigmoid notch and a superior osteotomy cut was planned below the zygomatic arch as the ankylotic fusion extended towards the sigmoid notch. To correct the chin deviation, genioplasty was planned which involved shifting the chin towards the left side.

In accordance with the aforementioned treatment plan, cutting guides were fabricated for the bilateral ankylotic masses as well as for the genioplasty. Furthermore, customized components for the temporomandibular joint (TMJ) were created, consisting of two distinct parts: the fossa component and the ramus-condyle unit. The fossa component was fabricated using ultra-high molecular weight polyethylene (UHMWPE), while the ramus-condyle unit was composed of titanium.

## 4 Surgery

**Fig. 2:**
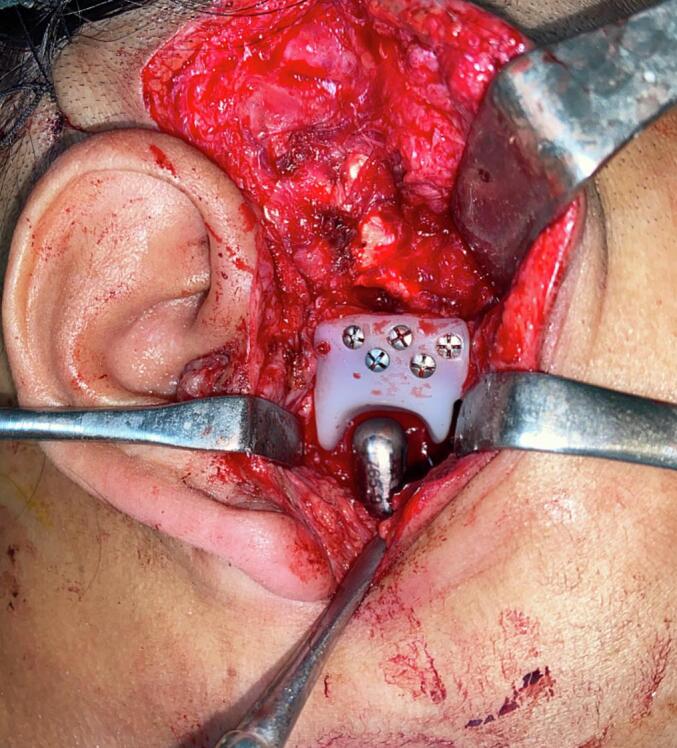
Intraoperative view illustrating a custom alloplastic temporomandibular joint (TMJ) fossa and condyle.

The patient was positioned supine for the procedure. Due to the absence of mouth opening, the anesthetist faced difficulty with intubation, necessitating fiberoptic-assisted nasotracheal intubation. Parts preparation was performed, followed by the implementation of an extended preauricular Al-Kayat Bramley incision and a standard submandibular incision. The ankylotic mass was explored and meticulously removed using cutting guides in a piecemeal fashion, creating a two-cm gap. The fossa component was securely fixed to the zygomatic arch using five screws on right side and six screws on left side, while the ramus-condyle unit was stabilized to the ramus using seven screws on the right side and eight screws on the left side, thereby achieving successful TMJ reconstruction (Fig. 2). To prevent a recurrence, the abdominal fat pad was harvested from the hypogastric region and interposed around the alloplastic condyle and fossa.

**Fig. 3:**
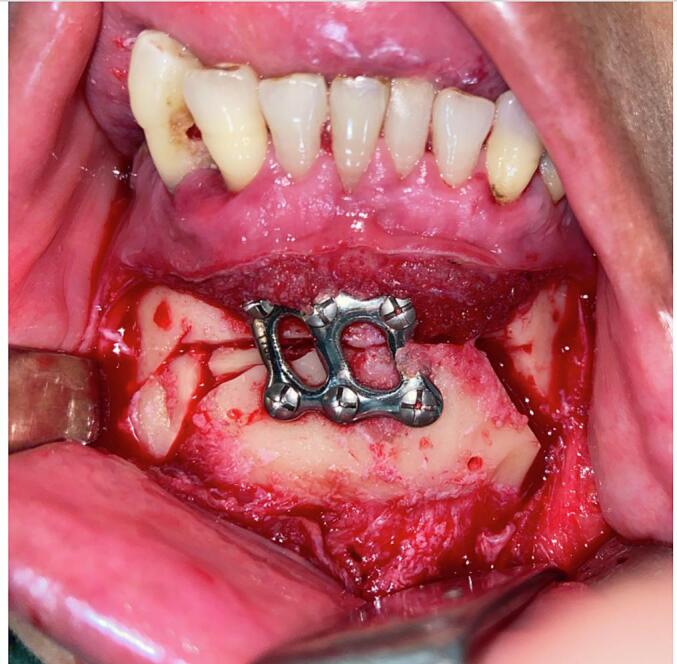
Intraoperative view depicting the performance of genioplasty, where the chin was repositioned to the left side and stabilized using a chinplate.

An intraoral vestibular incision was made in the lower anterior vestibule. A mucoperiosteal flap was elevated, and an osteotomy cut was performed. A segment of bone measuring nine millimeters was removed from the left side of the chin. Subsequently, the chin was shifted laterally by nine millimeters to the left side. The bone harvested from the left side of the chin was utilized as a graft and interposed on the right side. The chin was secured in place using a chin plate and six screws. (Fig. 3).

**Fig. 4:**
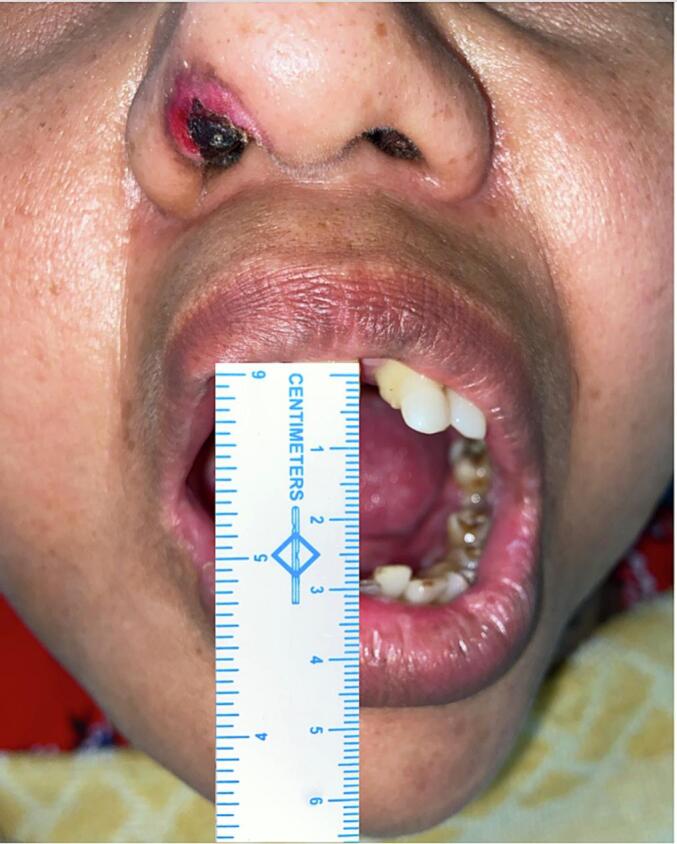
Postoperatively, the patient achieved a mouth opening of 30 mm.

**Fig. 5:**
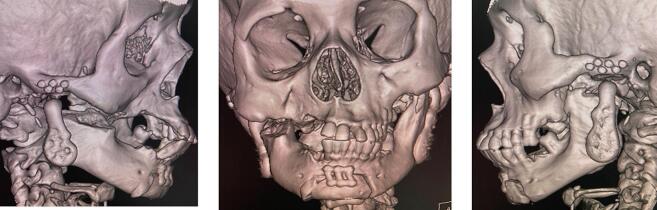
Postoperative Volumetric Rendering Technique (VRT) images displaying bilateral custom alloplastic temporomandibular joint (TMJ) replacements and genioplasty.

Hemostasis was achieved and primary closure was done after thorough irrigation. The patient was extubated. A postoperative mouth opening of 30 mm was achieved (Fig. 4). Detailed post-surgical instructions were provided to the patient and analgesics and antibiotics were prescribed. A postoperative NCCT of the face confirmed the successful bilateral custom alloplastic TMJ reconstruction and genioplasty (Fig. 5).

## 5 Discussion

The objective of this case was to manage bilateral TMJA in adult patients utilizing bilateral custom alloplastic TMJ prostheses in combination with genioplasty. Despite the availability of multiple treatment options, preference was given to custom alloplastic TMJ replacement to minimize the potential complications associated with autogenous grafts. A study conducted by Wolford and Mercuri in 2014 observed that custom joints exhibited no complications in a cohort of 56 patients [7]. Alloplastic TMJ prostheses provide a biomechanical solution rather than a biological one for TMJ-related issues [7]. Attaining satisfactory mouth opening is recognized as the primary and critical objective in the surgical management of TMJA. Multiple studies have consistently demonstrated the achievement of adequate mouth opening through the utilization of alloplastic TMJ [ 4,7,9,10].

An abdominal fat pad was utilized as an interposition material surrounding the custom alloplastic TMJ. Recurrence of TMJA due to heterotopic bone formation has been documented in approximately 50 % of cases [11]. To mitigate the occurrence of such complications, it is advisable to employ the placement of an abdominal fat pad surrounding the alloplastic TMJ [12].

The ramus-condyle unit of the custom alloplastic temporomandibular joint (TMJ) is fabricated using titanium, while the fossa component is composed of ultra-high molecular weight polyethylene (UHMWPE). Wear and tear, as well as hypersensitivity reactions to the alloplastic TMJ, can occur; however, the incidence is lower in metal on UHMWPE compared to metal-on-metal configurations [13]. Furthermore, the wear and tear of the alloplastic temporomandibular joint (TMJ) is minimal due to its non-load-bearing nature. During the surgical procedure, the temporalis and lateral pterygoid muscles are disinserted, further contributing to the reduced stress and strain on the alloplastic TMJ [13].

The remarkable strength of this surgical case lies in the successful restoration of normal temporomandibular joint (TMJ) function following an extended period of ankylosis spanning three decades, without encountering any complications. Importantly, no weaknesses or limitations were identified in this specific case.

## 6 Conclusion

Custom alloplastic TMJ prostheses are considered an optimal treatment modality for reconstructing the TMJ in adult patients with TMJ ankylosis.

## 7 Patient perspective

The patient expressed great satisfaction and appreciation to the surgical team for their intervention. After a prolonged period of 30 years, she successfully regained the ability to open her mouth, have regular meals and communicate effectively. Notably, the treatment process was relatively short and free from any complications. As a result, the patient experienced a substantial improvement in her quality of life.

## CRediT authorship contribution statement

Dr. Manoj Adhikari: planning, study concept or design, operating surgeon, manuscript writing and editing, etc.

Dr. Chandan Upadhyaya: Contributions: Treatment planning, study concept or design, operating surgeon, etc.

Dr. Kanistika Jha: Contributions: study concept or design, manuscript writing and editing, etc.

Dr. Galav Adhikari: Contributions: Treatment planning, manuscript editing, etc.

This case was operated at Sri Birendra Hospital, a tertiary care center located in Kathmandu, Nepal. It's a government hospital.

The surgery was conducted by two consultant Oral and Maxillofacial Surgeons, Dr. Manoj Adhikari and Dr. Chandan Upadhyay.

## Source of funding

This case did not receive any specific grant from funding agencies in the public, commercial or not-for-profit sectors.

## Ethical approval

It is our routine standard surgical procedure so ethical clearance was not required.

## Consent

Written informed consent was obtained from the patient for the publication of this case report and accompanying images. A copy of the written consent is available for review by the Editor-in-Chief of this journal on request.

## Research registration

This case report does not include any ‘first in man’ studies, so, registration was not required.

## Provenance and peer review

Not commissioned, externally peer-reviewed.

## Guarantor

Dr. Manoj Adhikari, Lecturer and Consultant Oral and Maxillofacial Surgeon, Nepalese Army Institute of Health Sciences, College of Medicine, Kathmandu, Nepal.

## Declaration of competing interest

The authors declare that there is no conflict of interest regarding the publication of this paper.

## Figures and Tables

**Supplementary Fig. 6:**
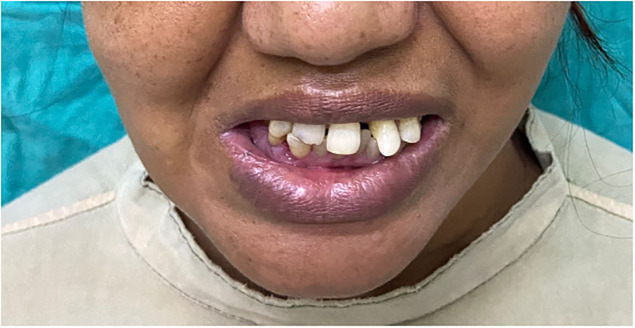
Preoperative view illustrating nil mouth opening.

**Supplementary Fig. 7:**
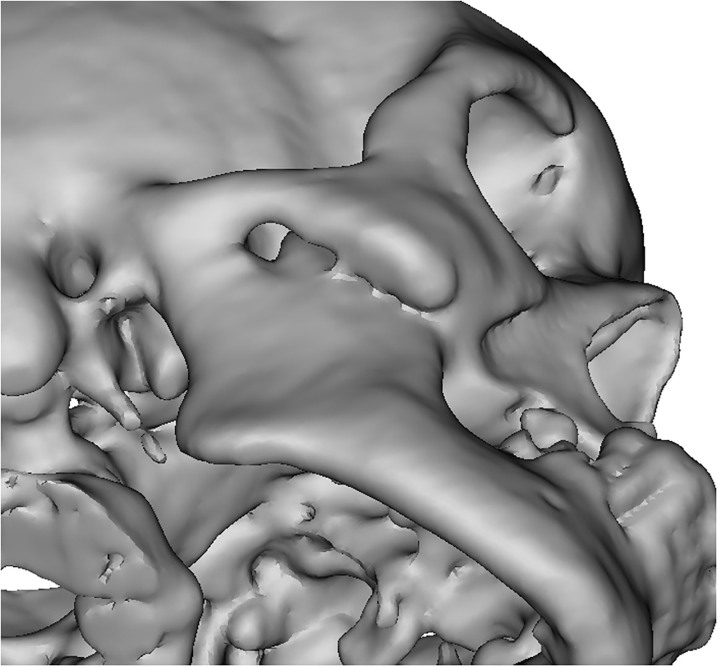
Volumetric Rendering Technique (VRT) image of the right side revealing the complete fusion of the entire ramus with the maxilla.

**Supplementary Fig. 8:**
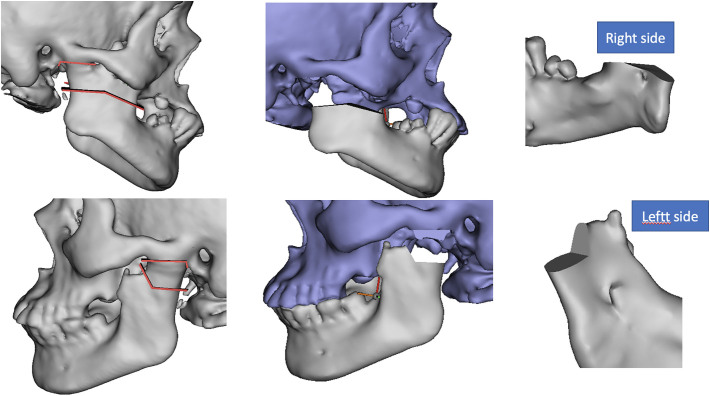
Planned osteotomy cuts made on both temporomandibular joint (TMJ) regions.

**Supplementary Fig. 9:**
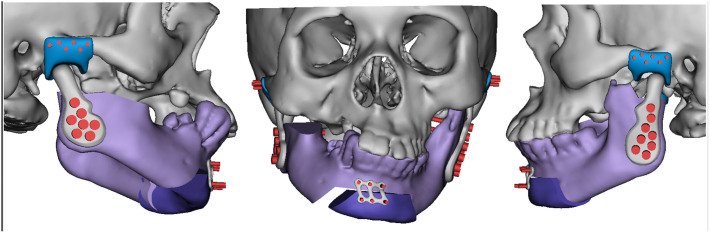
Total Facial correction design.

**Supplementary Fig. 10:**
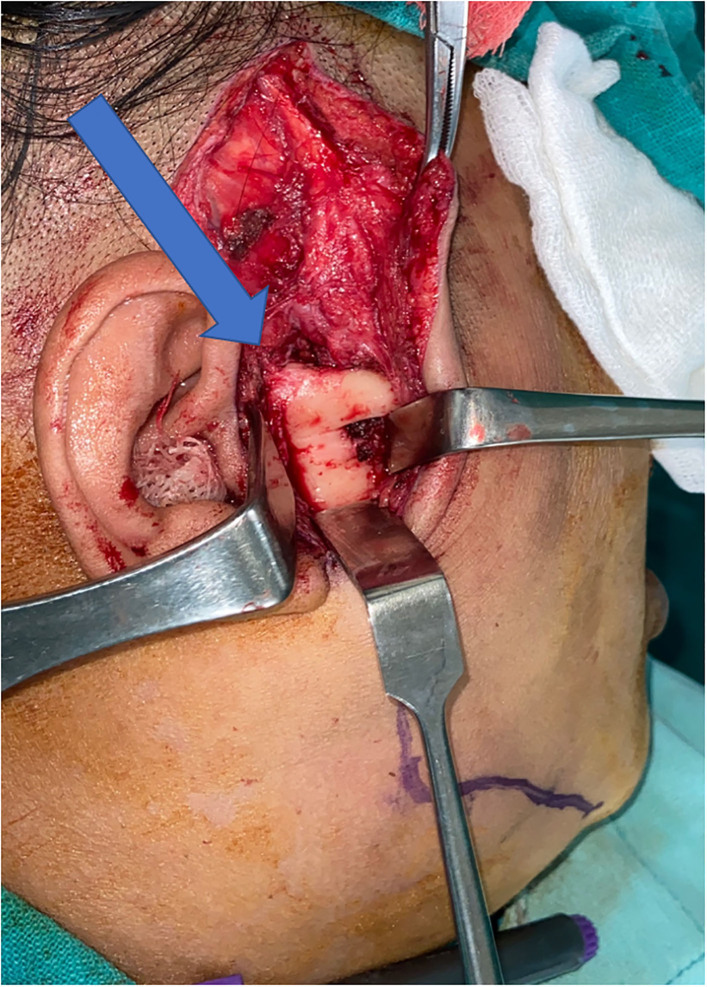
Intraoperative view illustrating the presence of an ankylotic mass in both temporomandibular joint (TMJ) regions.

**Supplementary Fig. 11:**
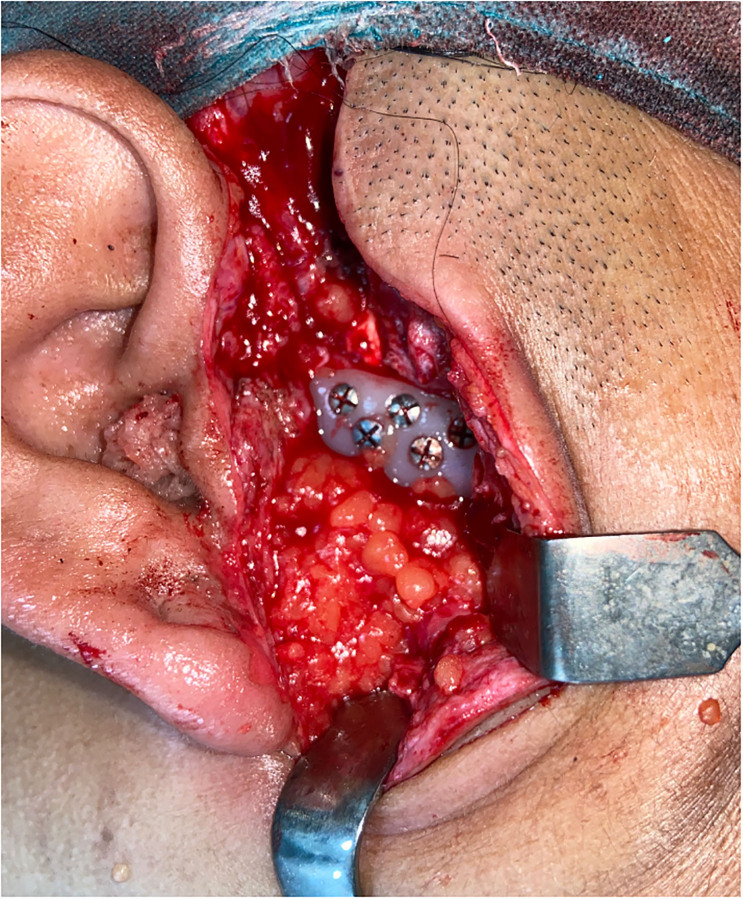
Intraoperative view demonstrating the utilization of abdominal fat pad interposition within the temporomandibular joint (TMJ) region.

**Supplementary Fig. 12:**
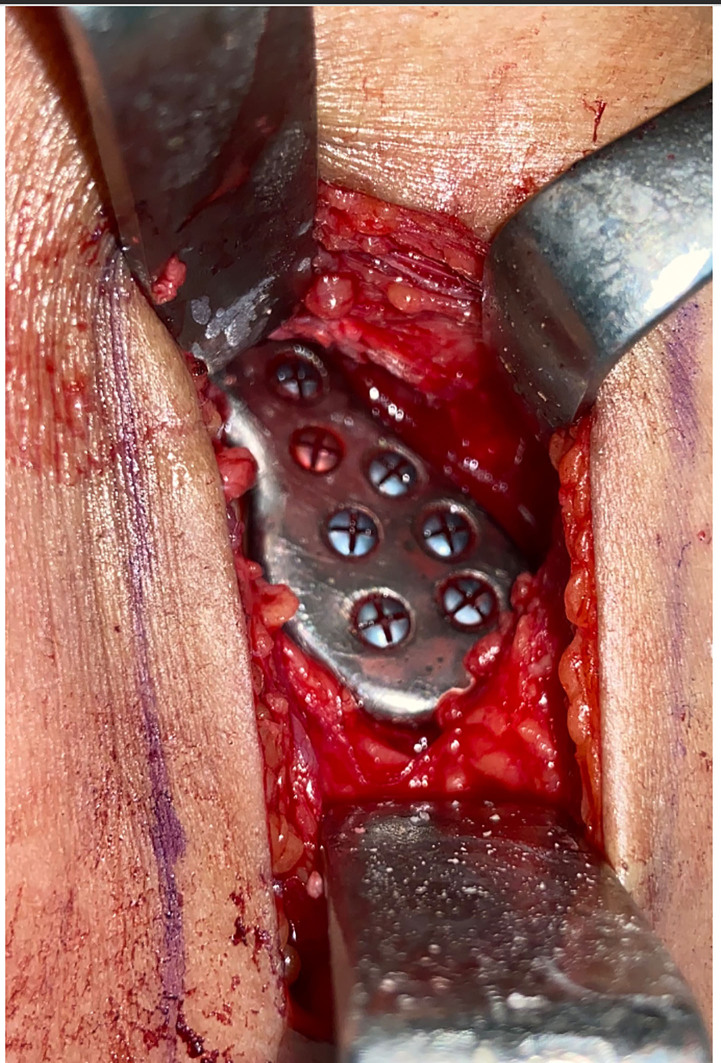
Intraoperative view displaying the ramal segment of the alloplastic temporomandibular joint (TMJ).
